# Hydranencephaly in a Newborn: A Case Report and a Review of the Literature

**DOI:** 10.7759/cureus.75435

**Published:** 2024-12-10

**Authors:** Khalil Toumi, Kamal Chafiq, Fatima Ezzahra Khayi, Abdellatif Daoudi

**Affiliations:** 1 Neonatology, Souss Massa University Hospital Center, Agadir, MAR

**Keywords:** brain imaging, congenital brain malformations, etiopathogenesis, hydranencephaly, prenatal diagnosis

## Abstract

Hydranencephaly (HE) is a severe and isolated malformation affecting the cerebral mantle. In this condition, the cerebral hemispheres are entirely or almost entirely absent, replaced by a membranous sac filled with cerebrospinal fluid, while the midbrain is usually preserved. Although HE is a relatively rare brain disorder, the differential diagnosis must include conditions such as severe hydrocephalus, porencephalic cysts, and alobar holoprosencephaly. This condition also raises ethical concerns, particularly regarding the criteria for surgical treatment. This case report, accompanied by a review of the literature, discusses the etiopathogenic and diagnostic aspects of HE, as well as its management, illustrated by the antenatal diagnosis of a newborn with HE, confirmed by brain MRI at four days of age.

## Introduction

Hydranencephaly (HE) is a rare congenital malformation where the cerebral hemispheres are absent and replaced by a large cavity filled with cerebrospinal fluid [1]. The pathogenesis is attributed to a vascular accident, likely caused by bilateral occlusion of the cerebral arteries occurring in utero, affecting fewer than one in 10,000 newborns [2,3]. Turnbull first described it in 1904 [4]. Spielmeyer introduced the term itself in 1905 and reported a case of this malformation in twins [5]. Bettinger referenced the condition in 1940 followed by Hamby, Krauss, and Beswick in 1950 [5]. However, similar clinical manifestations had already been documented earlier by Cruveilhier in his work *Anatomie Pathologique du Corps Humain* [5].

Hydranencephaly is a severe anomaly often caused by bilateral occlusion of the internal carotid arteries in utero, typically during the second trimester of pregnancy. Its etiologies include infectious, toxic, genetic, or vascular factors, as well as complications such as maternal hypoxia or twin-to-twin transfusion syndrome. Diagnosis relies on advanced imaging techniques, particularly fetal ultrasound, which can reveal a large fluid-filled cavity with no visible cortex, later confirmed by MRI or CT after birth [6,7].

This condition may require surgical intervention, such as a ventriculoperitoneal shunt or choroid plexus coagulation (CPC) combined with ventriculostomy, to manage complications associated with intracranial hypertension. It is crucial to inform families about the available therapeutic options to enable them to make an informed decision. Comprehensive care, including physiotherapy, seizure management, nutritional support, and treatment of respiratory disorders, is essential to improve the quality of life for patients [5].

We present the case of a male newborn, born at term to non-consanguineous parents, from a poorly monitored pregnancy, diagnosed with hydranencephaly. The prenatal diagnosis was established late at 30 weeks of gestation and confirmed by a brain MRI performed on the fourth day of life.

## Case presentation

**Figure 1:**
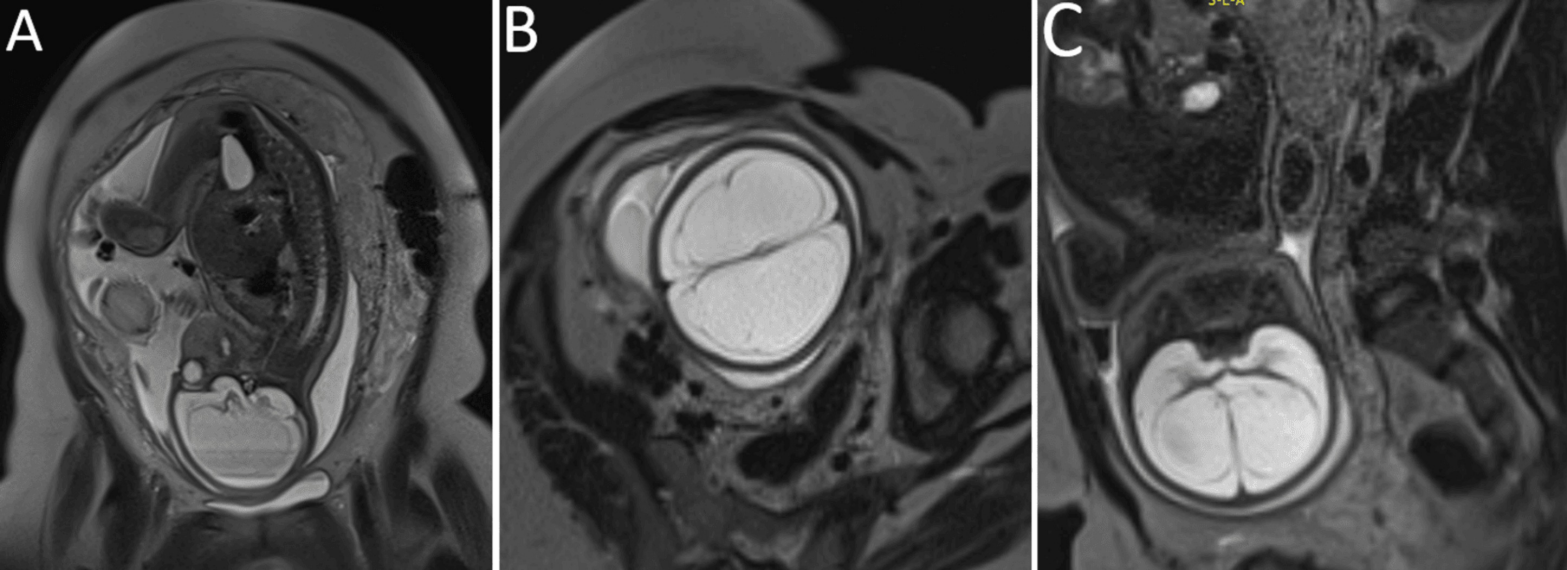
Pelvic MRI, performed in sagittal T2 (A), axial T2 (B), and coronal T2 (C) views, reveals a twin pregnancy The first fetus shows atrophy, indicating a non-viable pregnancy. The living fetus presents with severe bilateral symmetrical hydrocephalus, leading to complete compression and thinning of the cerebral parenchyma.

The patient was a male newborn weighing 2110 grams, delivered by cesarean section at 32 weeks of gestation to a 36-year-old multiparous mother from a twin pregnancy complicated by in-utero fetal demise. The pregnancy was poorly monitored, including a pelvic MRI at 30 weeks that revealed a twin pregnancy. The first fetus showed atrophy, indicating a non-viable pregnancy. The living fetus presented with severe bilateral symmetrical hydrocephalus, leading to complete compression and thinning of the cerebral parenchyma (Figure 1). The parents were non-consanguineous, with no family history of malformations and no exposure to drugs, carbon monoxide, infections, tobacco, or cocaine during the pregnancy.

**Figure 2:**
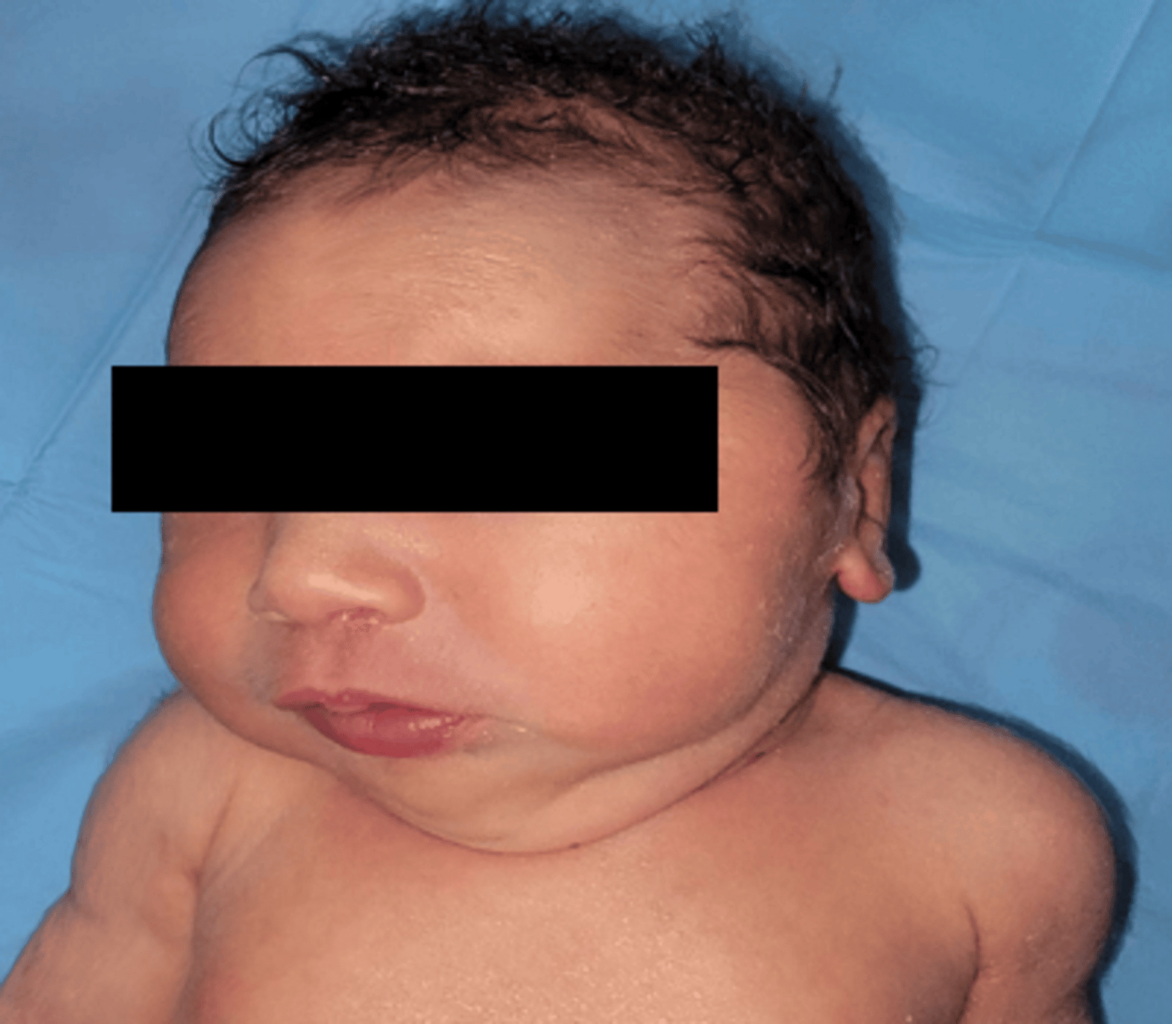
Newborn’s face showing microcephaly

On clinical examination, the Apgar score was 7/10 at 1’ and 8/10 at 5’. Microcephaly was noted, with a head circumference of 24 cm, corresponding to more than three standard deviations below the mean, with no other craniofacial anomalies observed. The newborn exhibited generalized hypotonia, but spontaneous reflexes such as sucking, swallowing, crying, and spontaneous limb movements were normal at birth. The cardiovascular and respiratory systems were clinically normal (Figure 2).

**Figure 3:**
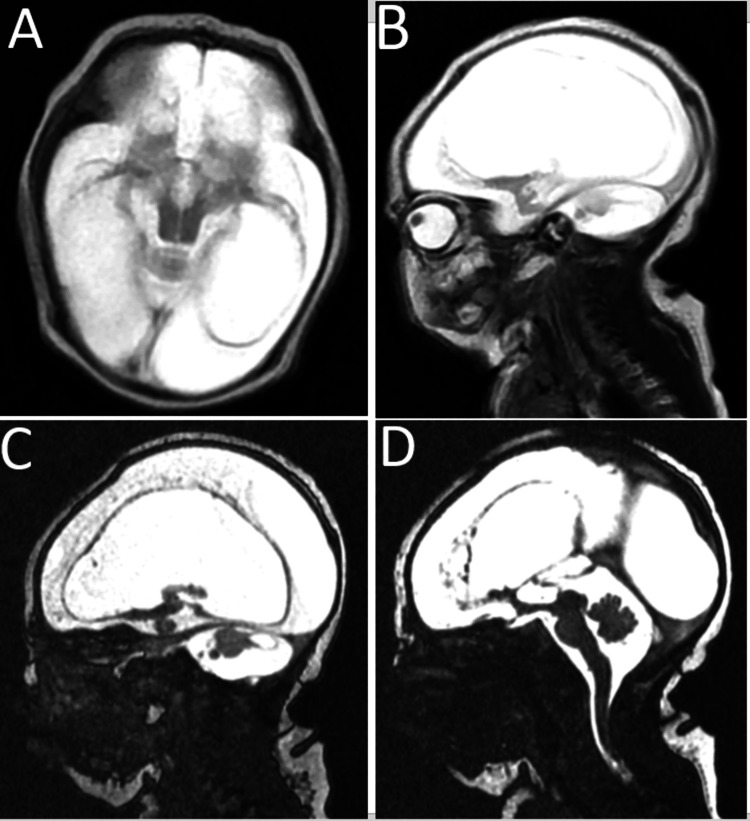
The MRI of the brain Axial (A) and sagittal (B) slices of a T2-weighted brain MRI show that the cerebral parenchyma and basal ganglia are almost entirely replaced by fluid isointense to CSF, with the persistence of a thin cortical rim. Sagittal image (C) shows atrophied cerebellar hemispheres with posterior cystic lesions containing fluid isointense to CSF. Sagittal image (D) shows a brainstem and vermis with normal morphology and signal.

The brain MRI showed a diffuse enlargement of the subarachnoid spaces, both supratentorial and infratentorial. The cerebral parenchyma and basal ganglia are almost entirely replaced by fluid that is isointense with CSF on all sequences, with only a thin cortical rim and a few bilateral temporopolar parenchymal islands remaining. The brainstem and vermis displayed normal morphology and signal (Figure 3). Renal and bladder ultrasound revealed no malformations.

The newborn was sent to the neurosurgery department, where the parents were informed about the prognosis and possible complications. They were also provided with psychological support and scheduled for regular follow-up consultations to closely monitor and manage any emerging complications.

## Discussion

Hydranencephaly is a rare congenital malformation characterized by the absence of cerebral hemispheres, which are replaced by a large cerebrospinal fluid-filled cavity. This condition is often associated with in-utero vascular accidents, although its etiology is multifactorial, leading to severe complications and a frequently poor prognosis [1,2]. This is a brain anomaly occurring in fewer than one in 10,000 births. No distinction exists between the sexes [3].

Bettinger credited Cruveilhier with providing the first description of HE, documented between 1829 and 1835 [5]. Cruveilhier described two distinct forms: hydrocephalic anencephaly and anencephaly with a complete absence of the cranial vault [5]. Later, in 1905, Spielmeyer first introduced the term "hydranencephaly" while studying this anomaly in dichorionic twins [5].

The etiopathogenesis of HE is generally caused by the occlusion of cerebral arteries above the supraclinoid level, resulting in the absence of cerebral hemisphere development [7]. Although various factors can disrupt the normal development of the vascular network, identified causes include intrauterine infections such as toxoplasmosis and various viruses (enterovirus, adenovirus, cytomegalovirus, herpes simplex virus, Epstein-Barr virus, and respiratory syncytial virus). Exposure to toxic substances, such as tobacco, cocaine, estrogens, and sodium valproate, has also been reported [5]. Finally, HE has been reported in monochorionic twin pregnancies, where the death of one twin could lead to vascular exchanges [8].

Hydranencephaly frequently manifests with normal fetal movements during pregnancy. At birth, newborns may present with a normal or increased head circumference, linked to the continuous production of cerebrospinal fluid. Although overt neurological signs are generally absent at birth, subtler symptoms such as hypotonia or feeding difficulties may quickly emerge [5]. This congenital malformation is primarily diagnosed through prenatal ultrasound, typically during the second trimester of pregnancy. Characteristic sonographic features include the absence of cerebral hemispheres, replaced by a homogenous echogenic structure occupying the supratentorial space, while the brainstem and cerebellum remain intact. The diagnosis can be confirmed through additional examinations, such as MRI or post-mortem analysis [9].

Hydranencephaly must be differentiated from extreme hydrocephalus, alobar holoprosencephaly, and porencephaly as part of the differential diagnosis. On ultrasound, HE appears as a large cystic mass without cerebral cortex, distinguishing it from other conditions [5]. It can be associated with various anomalies, including cerebellar involvement, ocular malformations, intracranial calcifications, renal dysplasia, and genetic syndromes such as Fowler syndrome, which is linked to mutations in the FLVCR2 gene. It is also described in complex contexts, such as polymicrogyria, holoprosencephaly, and chromosomal anomalies [10].

This malformation leads to severe complications such as developmental delays, seizures, frequent respiratory infections, respiratory distress, and poor growth, which are often fatal. It is also associated with cerebral palsy and gastroesophageal reflux, sometimes requiring prolonged ventilation [11]. Additionally, endocrine disturbances such as hypothyroidism, hypocortisolism, and panhypopituitarism are also frequently observed in these patients [12].

In our patient, the prenatal diagnosis of the malformation was established late, at 30 weeks of gestation, due to inadequate pregnancy follow-up, making therapeutic termination impossible. Hydranencephaly was confirmed postnatally by a brain MRI performed on the fourth day of life. An earlier diagnosis could have allowed for more tailored management; however, in Morocco, therapeutic termination of pregnancy remains a complex issue, confronted with ethical, moral, legal, and religious considerations.

The treatment of HE focuses on managing symptoms and associated complications, as the prognosis is generally poor, with in-utero death or death within the first year of life. In cases of prenatal diagnosis, therapeutic abortion may be proposed to limit maternal morbidity after thorough discussions with the family. Care includes managing hydrocephalus with shunt placement, treating seizures with antiepileptic medications, performing tracheostomy in cases of respiratory failure, and providing physical therapy and nutritional support to improve the patient's overall condition [5]. Ventriculoperitoneal shunting is commonly used to treat hydranencephaly by regulating head circumference, although this procedure can lead to complications such as infections. As an alternative, choroid plexus cauterization is considered to avoid shunt-related issues [13].

The prognosis for patients with HE is generally poor, with a limited life expectancy and frequent deaths occurring within the first weeks or year of life, often due to complications [14]. Nevertheless, rare cases of prolonged survival have been reported, with some patients living up to 20 or 22 years, though such cases are generally marked by a significant absence of neurological improvement [5,15,16].

## Conclusions

Hydranencephaly is a rare congenital malformation characterized by significant variability in its clinical manifestations, depending on the severity of the anomaly. In most cases, prenatal ultrasound is sufficient to establish the diagnosis. An MRI or intrauterine CT can complement the ultrasound assessment but should not be considered first-line diagnostic tools. Despite advancements in imaging techniques, managing this condition remains challenging due to the ethical and clinical dilemmas associated with the therapeutic termination of pregnancy. A thorough understanding of the etiopathogenic mechanisms of HE, combined with a multidisciplinary approach, is essential to optimize the care of affected children and provide appropriate support to their families.
